# The effects of trace metal impurities on Ga-68-radiolabelling with a tris(3-hydroxy-1,6-dimethylpyridin-4-one) (THP) chelator†

**DOI:** 10.1039/c9ra07723e

**Published:** 2019-11-14

**Authors:** Ruslan Cusnir, Andrew Cakebread, Margaret S. Cooper, Jennifer D. Young, Philip J. Blower, Michelle T. Ma

**Affiliations:** School of Biomedical Engineering and Imaging Sciences, King's College London, St Thomas' Hospital London SE1 7EH UK michelle.ma@kcl.ac.uk; Laboratory of Radiochemistry, Paul Scherrer Institute 5232 Villigen-PSI Switzerland; Mass Spectrometry Facility, King's College London Franklin Wilkins Building, 150 Stamford St London SE1 9NH UK

## Abstract

GMP-grade ^68^Ge/^68^Ga generators provide access to positron-emitting ^68^Ga, enabling preparation of Positron Emission Tomography (PET) tracers and PET imaging at sites that do not have access to cyclotron-produced radionuclides. Radiotracers based on tris(3-hydroxy-1,6-dimethylpyridin-4-one) (THP) chelators enable simple one-step preparations of ^68^Ga PET radiopharmaceuticals from pre-fabricated kits without pre-processing of generator eluate or post-purification. However, trace metal impurities eluted along with ^68^Ga could compete for THP and reduce radiochemical yields (RCY). We have quantified trace metal impurities in ^68^Ga eluate from an Eckert & Ziegler (E&Z) generator using ICP-MS. The metals Al, Fe, ^nat^Ga, Pb, Ti and ^nat^Zn were present in generator eluate in significantly higher concentrations than in the starting eluent solution. Concentrations of Fe and ^nat^Ga in eluate were in the range of 0.01–0.1 μM, Al, Zn and Pb in the range of 0.1–1 μM, and Ti in the range of 0.9–1.5 μM. To assess the ability of THP to chelate ^68^Ga in the presence of such metal ions, radiolabelling reactions were undertaken in which selected metal ions were added to make them equimolar with THP, or higher. Al^3+^, Fe^3+^, ^nat^Ga^3+^ and Ti^4+^ reduced RCY at concentrations equimolar with THP and higher, but at lower concentrations they did not affect RCY. Pb^2+^, Zn^2+^, Ni^2+^ and Cr^3+^ had no effect on RCY (even under conditions in which each metal ion was present in 100-fold molar excess over THP). The multi-sample ICP-MS analysis reported here is (to date) the most comprehensive and robust quantification of metal impurities in the widely used E&Z ^68^Ga generator. ^68^Ga from an E&Z generator enables near-quantitative radiolabelling of THP at chelator concentrations as low as 5 μM (lower than other common gallium chelators) without pre-processing. The combination of Al^3+^, Fe^3+^, ^nat^Ga^3+^ and Ti^4+^ in unprocessed ^68^Ga eluate is likely to decrease RCY of ^68^Ga radiolabelling if a lower amount of THP chelator is used, and future kit design should take this into account. To increase specific activities by using even lower THP concentrations, purification of ^68^Ga from trace metal ions will likely be required.

## Abstract 2

We have quantified trace metal impurities present in ^68^Ga generator eluant from the widely used Eckert & Ziegler ^68^Ga generator, and measured the effect of these metal impurities on ^68^Ga radiolabelling of a THP chelator.

## Graphical abstract

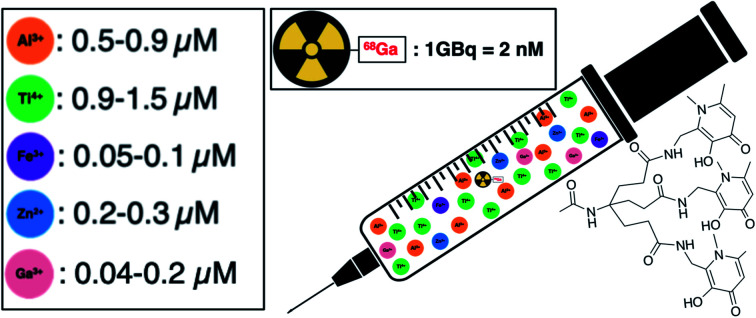

## Background

Molecular positron emission tomography (PET) imaging with radiolabelled receptor-targeted peptides and proteins has been transformative in management of cancer patients in clinical centres where it is available.^1,2^ Gallium-68 (^68^Ga) is a metallic radionuclide that emits positrons (*t*_1/2_ = 68 min, *β*^+^ 90%, *E*_max_ 1880 keV) suitable for diagnostic imaging with ^68^Ga-labelled chelator-peptide conjugates.^3^ The short half-life of ^68^Ga is compatible with the fast clearance from blood and rapid target localisation of ^68^Ga-labelled chelator-peptide conjugates. However, it also necessitates fast radiolabelling approaches.

One-step, kit-based radiolabelling protocols that provide a radiopharmaceutical in quantitative radiochemical yield (RCY), and in pyrogen-free, physiologically-compatible solutions are the ideal for ^68^Ga radiopharmaceuticals.^4,5^ Such kit-based radiosyntheses reduce the need for expensive infrastructure and highly trained personnel. Kit-based radiosynthesis protocols have been used for formulation of ^99m^Tc radiopharmaceuticals for almost five decades. These “one-step” kits consist of a single pre-fabricated vial that contains all non-radioactive components and reagents. For simple kit-based radiosynthesis of ^68^Ga radiopharmaceuticals to be realised, the nuclear medicine community needs chelators that bind ^68^Ga rapidly (<5 min) and quantitatively (RCY > 95%) at low chelator concentrations (μM range) and near physiological pH (6–7), preferably avoiding heating or other additional manipulations.

DOTA and HBED-CC chelators are used in the radiopharmaceuticals ^68^Ga-DOTA-TATE for imaging neuroendocrine tumours^6^ and ^68^Ga-HBED-PSMA for imaging prostate tumours,^7^ respectively. To date, these chelators have typically required pre-processing of ^68^Ga generator eluate to remove competing trace metal impurities and concentrate ^68^Ga^3+^, heating to reproducibly incorporate ^68^Ga^3+^ into the chelator, and post-synthetic purification to remove unreacted ^68^Ga^3+^ and physiologically-incompatible buffer components.^8–10^ Hence, they do not meet the ideal requirements of kit-based radiolabelling.

**Fig. 1:**
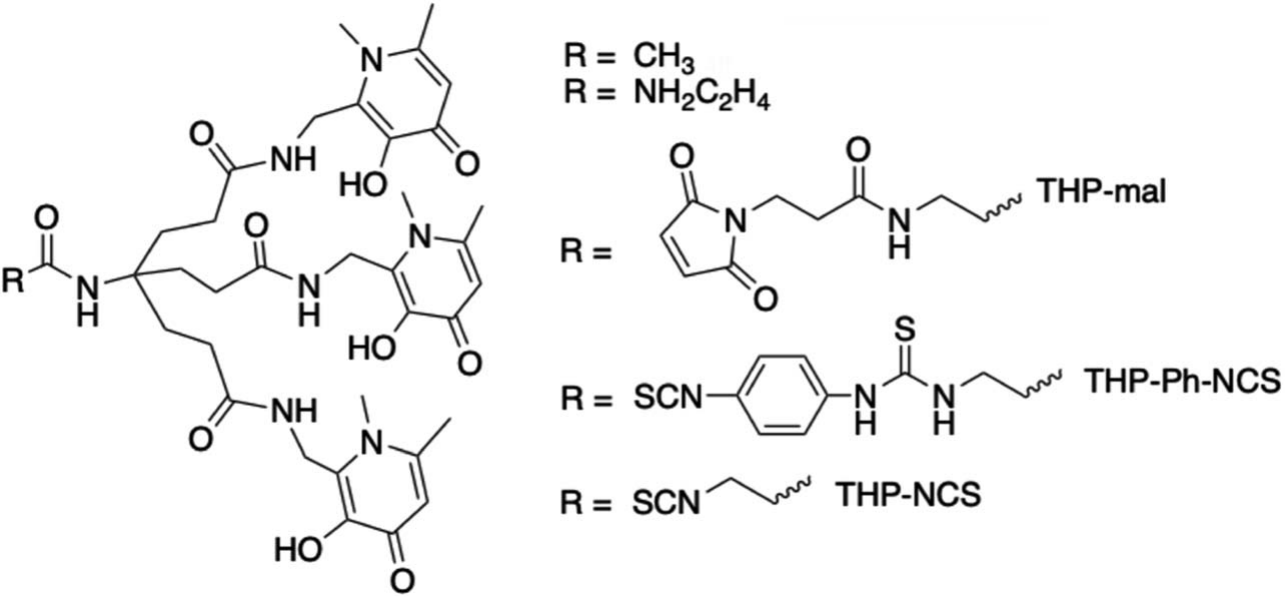
THP chelator (R = CH_3_) and its bifunctional chelator derivatives.

We have recently developed a THP (tris(3-hydroxy-1,6-dimethylpyridin-4-one)) chelator (Fig. 1) that rapidly and quantitatively incorporates ^68^Ga^3+^ at room temperature, neutral pH, and low chelator concentrations, giving a single species.^11–13^ It is consequently well-suited to kit-based ^68^Ga-radiopharmceutical synthesis, unlike other common gallium chelators such as DOTA and HBED-CC. THP has demonstrated superior radiolabelling properties compared to many other chelators, with higher RCYs achievable for THP at very low concentration compared to *e.g.* DOTA and HBED.^14^ THP has been attached to a number of peptides and proteins that target cancer cell surface receptors and the conjugates demonstrate favourable biodistribution and targeting properties in *in vivo* PET imaging studies.^5,11,15–19^ A THP bioconjugate, THP-PSMA, that targets the prostate specific membrane antigen (PSMA) has been evaluated recently for one-step kit-based radiolabelling with ^68^Ga.^5^ These resulting GalliProst™ kits can be radiolabelled in 2–5 min, simply by addition of unprocessed ^68^Ga generator eluate (typically 5 mL of 0.1 M aqueous HCl containing 170–270 MBq of ^68^Ga) to a vial containing THP-PSMA and sodium bicarbonate buffer.^5,16^ A new homologue of THP has been developed with further improved radiolabelling properties and affinity and selectivity for Ga^3+^.^13^

Currently, ^68^Ga is usually produced from pharmaceutical grade ^68^Ge/^68^Ga generators.^20,21^ In these generators, the parent radionuclide ^68^Ge (*t*_1/2_ = 271 days) is adsorbed on a stationary phase, typically TiO_2_. ^68^Ge decay yields ^68^Ga, which is normally eluted with an acidic solution (typically aqueous hydrochloric acid) as ^68^Ga^3+^. Frequent elutions and continuous use of acid can result in leaching of other metal ions from the generator stationary phase. These can significantly reduce radiolabelling efficiency due to competition with the radiometal ion for chelator binding.^22^ Additionally, the concentration of ^68^Ga^3+^ is very low: 1 GBq of carrier-free ^68^Ga contains only 9.8 pmol of ^68^Ga^3+^, equivalent to approx. 2 nM concentration in 5 mL of generator eluate. Most chelators do not exclusively bind a single metal ion, and so both the selectivity of a chelator and the purity of ^68^Ga eluate can influence the RCYs of these reactions. Some ^68^Ga chelators, such as HBED,^23^ NOTA^24^ and TRAP^24^ have demonstrated selectivity for Ga^3+^ over divalent metal ions.

THP ligands were originally developed as iron-sequestering chelators,^25,26^ and so as well as having high affinity for Ga^3+^, THP also has high affinity for Fe^3+^, Al^3+^ and other oxiphilic transition metal ions.^12,13,27–29^ The highest molar activity that we have achieved for a THP conjugate to date is 80 MBq nmol^−1^,^11,19^ equating to <0.1% ^68^Ga^3+^ occupancy of THP in these solutions. This is comparatively low compared to other radiometallated conjugates – for example, some DTPA- or DOTA-conjugates with ^111^In can achieve specific activities up to 1 GBq nmol^−1^,^30^ and TRAP-conjugates with ^68^Ga can achieve over 4.8 GBq nmol^−1^, although the latter is obtained under reaction conditions that result in <70% RCY.^31^ It is likely that competition from interfering metal ions in ^68^Ga generator eluate limits achievement of higher molar activities for THP.

The objectives of this study were to (i) quantify trace metal impurities in ^68^Ga eluate from an Eckert & Ziegler generator and (ii) evaluate the competition effects of the most common contaminant metal ions on ^68^Ga radiolabelling with THP.

## Methods

### Reagents

Sterile ultrapure 0.1 M hydrochloric acid aqueous solution for elution of ^68^Ga from ^68^Ge/^68^Ga Eckert & Ziegler generator was supplied by ABX GmbH (Radeberg, Germany). One-year old generators (yielding 350–450 MBq) were used in this study. Lead, titanium and zinc standard solutions (1000 mg L^−1^) for AAS were purchased from VWR. Gallium and nickel standards for AAS (TraceCERT TM, 1000 mg L^−1^) were purchased from Sigma-Aldrich. Chromium (as Cr^3+^) solution for ICP (1000 mg L^−1^) was purchased from Fisher Chemical. Aluminium standard for ICP (1000 mg L^−1^) was purchased from Fluka. Iron AAS standard solution (Specpure®, 1000 mg L^−1^) was from Alfa Aesar. Sodium chloride ultrapure bioreagent was obtained from Fisher Scientific. (+)-Sodium l-ascorbate BioXtra was obtained from Sigma-Aldrich. Hydrochloric acid 20% Primar for trace metal analysis from Fisher Chemical was used for conditioning SCX columns. THP was synthesised according to a previously described procedure.^32^ Stock solutions of THP in water (UltraPure™ distilled water from Invitrogen) were stored at −20 °C. Instant thin layer chromatography strips impregnated with silica gel (iTLC-SG) were obtained from Agilent Technologies and used to determine RCYs. Ethanol and acetone were of HPLC-grade.

### Determination of trace metals in ^68^Ga generator eluate

^68^Ga^3+^ was eluted from an E&Z ^68^Ge/^68^Ga generator with ultrapure HCl (5 mL, 0.1 M) in a single fraction. Eluates were allowed to decay for at least 24 h prior to analysis by ICP-MS. Eluent (ultrapure 0.1 M HCl) was also analysed as a blank. All samples were stored in polypropylene tubes (Elkay, cat. no. 2086) with low trace metal content. The concentration of trace metals was determined on a PerkinElmer NexION 350D inductively coupled plasma mass spectrometer running Syngistix v1.0 software (London Metallomics Facility, King's College London, UK). The acquisition mode included 5 replicates averaged to give reported values for ^27^Al, ^137^Ba, ^52^Cr, ^65^Cu, ^56^Fe, ^69^Ga, ^55^Mn, ^60^Ni, ^23^Na, ^208^Pb, ^45^Sc, ^118^Sn, ^47^Ti, ^51^V, ^66^Zn and ^68^Zn. The dwell time was 50 ms per isotope, with 18 L min^−1^ main argon flow, 1.2 L min^−1^ auxiliary argon flow, 0.97 L min^−1^ nebuliser argon flow (optimised daily), 1600 W RF power, 0.2 mL min^−1^ sample flow, and KED cell mode with 4.2 mL min^−1^ helium flow. Concentrations of the individual isotopes, ^66^Zn and ^68^Zn, were determined by ICP-MS. ^nat^Zn was calculated based on natural abundance of ^66^Zn (27.9%). ^68^Zn arising from decay of ^68^Ga (^decay68^Zn) was calculated by subtracting naturally occurring ^68^Zn (18.75%) from ^68^Zn determined by ICP-MS.

### Competition experiments

To evaluate competition effects of trace metals, THP (5 μM) in sodium bicarbonate buffer was radiolabelled with ^68^Ga in the presence of additional Al^3+^, Fe^3+^, ^nat^Ga^3+^, Ti^4+^, Pb^2+^, Zn^2+^, Ni^2+^ and Cr^3+^ (0.05–500 μM), using the following solutions:

#### THP solutions

An aliquot of THP solution (54 μM, 28 μL) was added to an aliquot of sodium bicarbonate solution (66–88 μL, 0.5 M, pH 8.35) to provide 5 μM concentration of chelator in the final reaction mixture. As the metal ion standards contained different amounts of acid, the volume of sodium bicarbonate (plus a 10% excess) was separately calculated for each radiolabelling, in order to neutralise 0.1 M HCl (in ^68^Ga^3+^ eluate) and the additional H^+^ from trace metal stock solutions.

#### ^68^Ga + added metal ion solutions

^68^Ga was eluted from an E&Z ^68^Ge/^68^Ga generator with 5 mL of 0.1 M HCl. An aliquot of ^68^Ga^3+^ eluate (2–6 MBq, 70–90 μL) was spiked with a solution containing a single trace metal (Al, Fe, Ga, Ti, Pb, Zn, Ni and Cr), to provide concentrations of trace metal ranging from 50 nM to 500 μM (50 nM, 500 nM, 5 μM, 50 μM, and 500 μM) in the final reaction mixture.

#### Radiolabelling reactions

The solutions containing ^68^Ga and added trace metal ion were added to the THP solutions. The reaction mixture was made up to 300 μL total volume (using 50–116 μL of 0.1 M HCl), and contained 1.1 μg (1.5 nmol, 5 μM) of THP. In each reaction solution, the final pH was 7. The reaction mixture was vortexed and incubated at room temperature for 10 min. Each radiolabelling experiment was replicated three times, with each experiment consisting of 5 technical replicates.

#### Radiolabelling reactions in the presence of ascorbate

Aqueous sodium l-ascorbate was added to a solution containing ^68^Ga^3+^ and Fe^3+^, prior to addition of THP. In these reaction solutions, the final concentration of ascorbate was 166 mM in 300 μL total volume. The pH and the concentrations of THP, ^68^Ga^3+^ and Fe^3+^ were as described above.

#### Determining RCY with iTLC

The RCY of [^68^Ga(THP)] was determined by iTLC using a mobile phase of 1 M ammonium acetate in water/methanol (50/50 v/v, pH 5.5). For colloidal and unreacted ^68^Ga: *R*_f_ < 0.1; for [^68^Ga(THP)]: *R*_f_ > 0.95. iTLC strips were imaged and quantified by digital autoradiography using a Cyclone® Plus phosphor imager and OptiQuant™ software (PerkinElmer).

### Radiolabelling with solutions ^68^Ga^3+^ pre-processed using cation exchange methodologies

Radiolabelling of THP was tested with ^68^Ga “pre-processed” on a SCX Bond Elut 100 mg cartridge using methods that use acidified NaCl,^33^ ethanol^9^ or acetone^10^ solutions to elute the trapped ^68^Ga^3+^ from the cation exchange resin. The amount of NaHCO_3_ required to neutralise processed ^68^Ga was determined experimentally by titration (in order to account for extra H^+^ released from the SCX column). Processed eluate (50 μL, 7–19 MBq ^68^Ga) was added to a solution of THP (5 μM in a final volume of 300 μL at pH 7). After incubation at RT for 10 min, RCY was determined by iTLC.

## Results

### Trace metal impurities

**Fig. 2:**
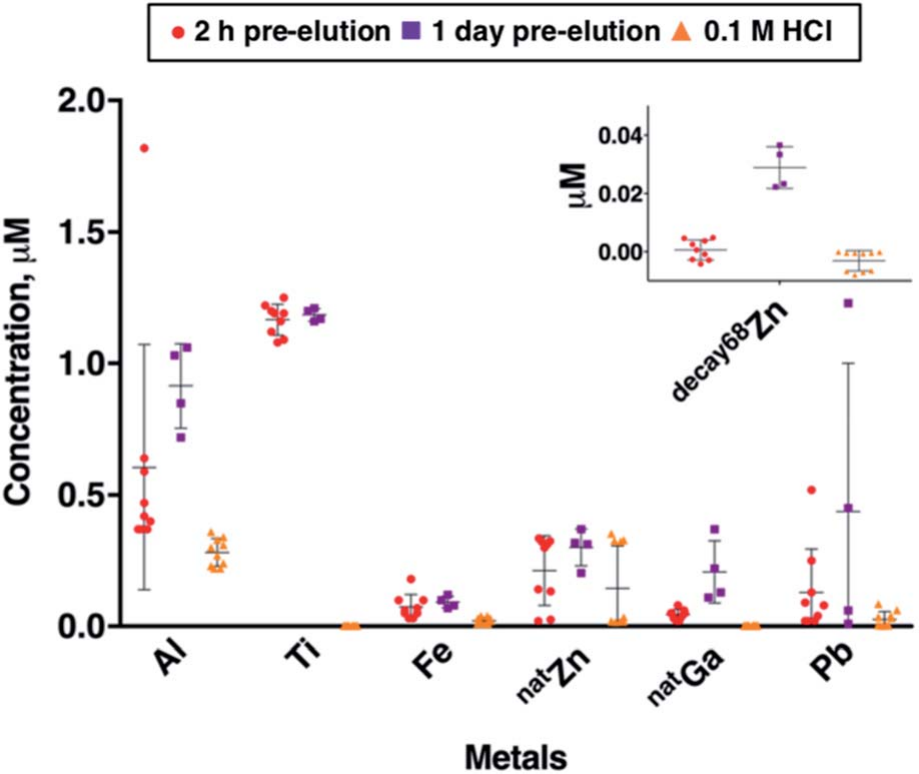
Concentrations of selected trace metals in ^68^Ga generator eluate measured by ICP-MS. Generator eluate (5 mL in 0.1 M HCl) was obtained from a single generator either 2 hours after a previous generator elution (sample set A, red circles, *n* = 9), or 1 day after a previous elution (sample set B, purple squares, *n* = 4). “Blank” eluent (0.1 M HCl solution from ABX Gmb) was also measured (yellow triangles, *n* = 10). These samples were collected within a period of 20 days, from a generator eluting 330–480 MBq. Error bars represent standard deviation of concentrations in the samples.

Using ICP-MS, we determined the concentrations of common trace metal impurities (Al, Cr, Cu, Fe, ^nat^Ga, Mn, Ni, Pb, Sc, Sn, Ti, V, ^66^Zn and ^68^Zn) in ^68^Ga eluate. The concentrations of trace metals are known to correlate with the amount of time between generator elutions.^14,22^ To probe this, we obtained generator eluate (5 mL in 0.1 M HCl) either (i) 2 hours after a previous generator elution (sample set A), or (ii) 1 day after a previous elution (sample set B). These samples were collected within a period of 20 days, from a generator eluting 330–480 MBq. The mean concentrations of Cr, Cu, Mn, Ni, Sc, Sn and V were all very low (below 10 nM) in both sample sets A and B, and in blank eluent solutions of 0.1 M HCl. We have therefore focused on transition and main group metals Al, Fe, ^nat^Ga, Pb, Ti and Zn (Fig. 2). The mean concentrations of metals Al, Fe, ^nat^Ga, Pb, Ti and ^nat^Zn were all higher in sample sets A and B than in eluent (blank) solution. These concentration differences between eluate and blank solution were statistically significant in all cases (*p* < 0.1, Table SI-1†), with the exception of the difference between mean ^nat^Zn concentrations in sample set A and blank solutions.

The metal present at highest concentrations in generator eluate was Ti. Mean Ti concentration was 1.17 ± 0.37 μM in sample set A, and 0.95 ± 0.53 μM in sample set B. There was no significant difference in mean Ti concentration between sample sets A and B. The high concentration of Ti in eluate has been previously observed by us and others: it arises from the solid phase titanium dioxide material (contained in a borosilicate glass column) on which ^68^Ge^4+^ is immobilised.^14,34^

The trivalent metal ions Al^3+^ and Fe^3+^ bind hydroxypyridinones with high affinities,^12,13,27–29^ and high concentrations of Al, Fe and non-radioactive ^nat^Ga (all likely to be present as trivalent cations) might therefore be expected to decrease specific activities of [^68^Ga(THP)] and its bioconjugates. Al was present in highest concentration, measuring 0.61 ± 0.47 μM in sample set A, and 0.91 ± 0.16 μM in sample set B. Al mean concentrations in blank eluent solutions (0.28 ± 0.05 μM) were relatively high compared to other metal impurities in blank eluent solutions. Fe mean concentrations were 0.07 ± 0.05 μM in sample set A and 0.09 ± 0.02 μM in sample set B. Whilst Al and Fe mean concentrations were both higher in sample set B compared with sample set A, neither of these observed differences were statistically significant. We observed that increased time between ^68^Ga generator elutions resulted in significantly increased amounts of non-radioactive ^nat^Ga in generator eluate. Mean ^nat^Ga concentration measured 0.04 ± 0.02 μM in sample set A, and 0.21 ± 0.12 μM in sample set B (mean difference = 0.16 μM, *p* = 1.4 × 10^−3^).

Many chelators used for ^68^Ga radiolabelling, including DOTA,^35^ have high affinity for divalent cations such as Zn^2+^ and Pb^2+^. Zn impurities arise from the eluent solution, leaching from generator components and decay of ^68^Ga. The presence of Zn (occurring as Zn^2+^) in generator eluate can compromise ^68^Ga radiolabelling of DOTA-based radiopharmaceuticals.^21,36,37^ In some clinical ^68^Ga radiosynthesis protocols, the generator eluate undergoes pre-processing to separate Zn^2+^ from ^68^Ga prior to biomolecule radiolabelling.^10,38^^68^Ga decays exclusively to ^68^Zn, and this contributes to the amount of Zn^2+^ present in eluate solution. Factors that directly correlate with the amount of ^68^Zn in generator eluate include the activity of the generator, and the amount of time between elutions of the generator. Here, we quantified (i) ^nat^Zn concentration (using ^66^Zn ICP-MS signal), and (ii) the amount of ^68^Zn arising from decay of ^68^Ga, “^decay68^Zn” (using ^68^Zn ICP-MS signal, and subtracting known proportions of ^nat68^Zn from this measurement).

Mean ^nat^Zn concentration measured 0.21 ± 0.13 μM in sample set A, and 0.30 ± 0.07 μM in sample set B and was higher in both sample sets compared to blank eluent solution (0.15 ± 0.16 μM), although this was only statistically significant for sample set B (mean difference = 0.15, *p* = 0.09). Mean ^decay68^Zn concentration measured 6.1 × 10^−4^ ± 3.4 × 10^−3^ μM in sample set A (in which samples were collected 2 hours after a previous elution), and 2.9 ± 0.7 × 10^−2^ μM in sample set B (in which samples were collected 1 day after a previous elution) (mean difference = 2.8 × 10^−2^, *p* < 10^−4^).

Mean eluate Pb concentrations in both sample set A (0.13 ± 0.16 μM) and sample set B (0.44 ± 0.56 μM) were higher than that observed in blank eluent solution (0.03 ± 0.03 μM), and there was high variability in Pb concentration between individual samples in both sample sets A and B. There was no statistically significant difference between Pb concentrations in sample sets A and B. It is likely that Pb content arises from lead structures used to the shield the generator. Others have also observed significant Pb content in generator eluate.^34^

### Changes in Al, Fe, Ga and Ti concentrations in eluate over six months

**Fig. 3:**
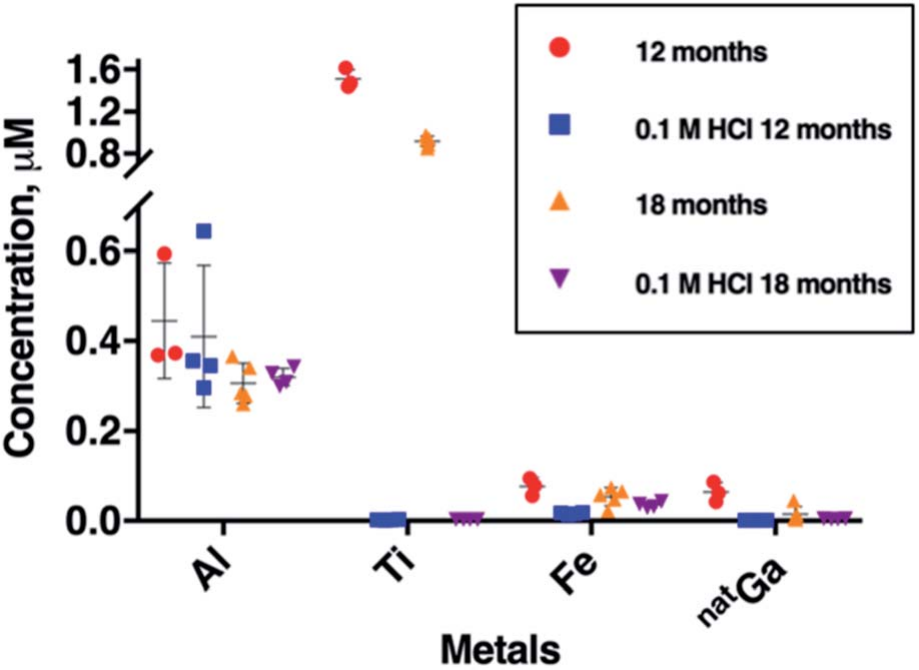
Concentrations of Al, Ti, Fe and ^nat^Ga in ^68^Ga eluate as a function of generator age. Two sets of eluate samples were collected from the same ^68^Ga E&Z generator, six months apart from each other. “Blank” eluent (0.1 M HCl solution from ABX Gmb) used in these two sets of samples was also measured. During this period, the generator was eluted 130 times. All eluates were sampled with a pre-elution window of 2 h. Error bars represent standard deviation of concentrations in the samples.

To further probe Al, Fe, Ga and Ti concentration in generator eluate, eluate samples were collected six months apart from each other, from a second E&Z generator (Fig. 3). These samples were all obtained 2 hours after a previous elution. During this six month period, between our two sample collection campaigns, the generator was eluted 130 times.

The most remarkable change was observed for mean Ti breakthrough, which decreased from 1.51 ± 0.09 μM to 0.92 ± 0.05 μM (mean difference = 0.59 μM, *p* = 1.8 × 10^−5^) within six months (Fig. 3 and Table SI-3†). Concentrations of Al and ^nat^Ga over this time also decreased. Mean Al concentration decreased from 0.45 ± 0.13 μM to 0.31 ± 0.05 μM (mean difference = 0.14 μM, *p* = 6.11 × 10^−2^) and mean ^nat^Ga concentration decreased from 0.064 ± 0.022 μM to 0.015 ± 0.018 μM (mean difference = 0.049 μM, *p* = 1.27 × 10^−2^). The mean concentration of Fe also decreased from 0.077 ± 0.020 to 0.054 ± 0.020 μM, however in our sample set, this was not statistically significant.

### The effect of metal ion impurities in ^68^Ga radiolabelling reactions with THP

To identify the effect of the presence of trace/interfering metal ions on radiolabelling THP with ^68^Ga, THP was radiolabelled with ^68^Ga in a solution containing a spike of each metal ion. We included the metals identified as being present in generator eluate at concentrations >10 nM (Al, Fe, ^nat^Ga, Pb, Ti and ^nat^Zn), as well as Ni and Cr, which are present in steel needles often used for preparation of radiopharmaceuticals. In these experiments, we used only a small amount of ^68^Ga eluate (2–6 MBq, 70–90 μL) to minimise the amount of interfering metal ions from other sources, such as the generator itself. We have also assumed that the metal ions are present as Al^3+^, Fe^3+^, ^nat^Ga^3+^, Pb^2+^, Ti^4+^, Zn^2+^, Ni^2+^ and Cr^3+^.

**Fig. 4:**
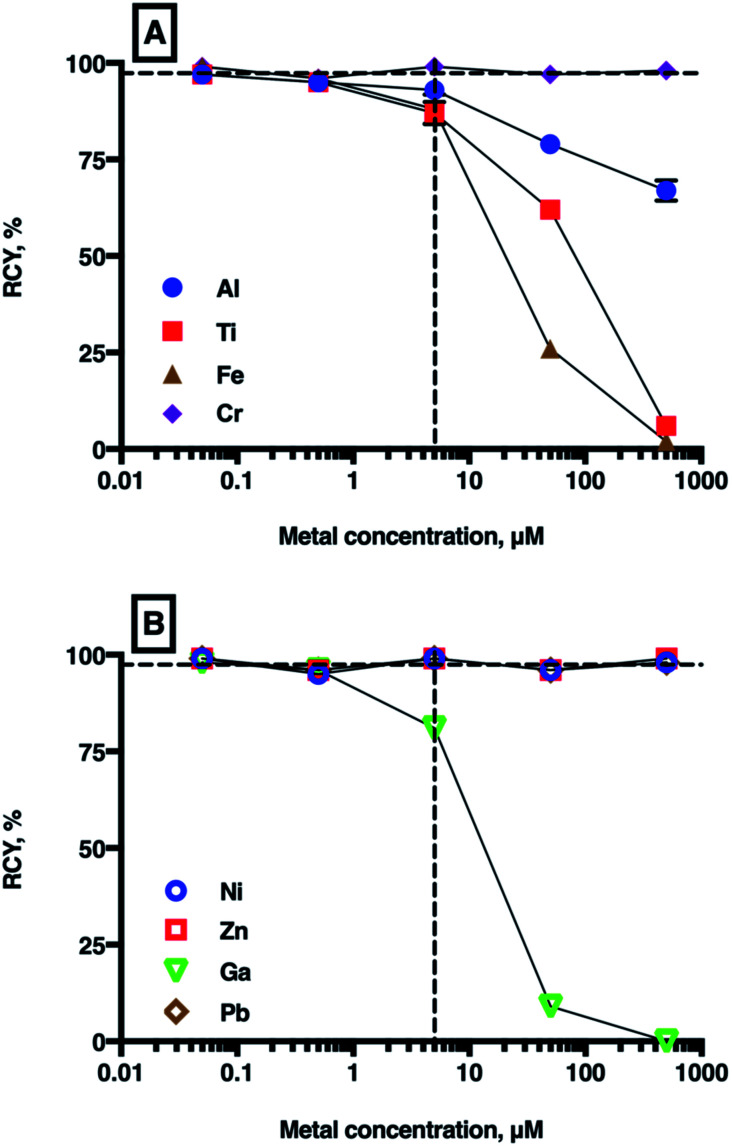
Mean RCYs of [^68^Ga(THP)] in the presence of increasing concentrations of metal ions ((A): Al^3+^, Ti^4+^, Cr^3+^, Fe^3+^ and (B): Ni^2+^, Zn^2+^, ^nat^Ga^3+^, Pb^2+^). The vertical dashed line indicates equimolar concentrations of chelator and metal ion (5 μM), and the horizontal dashed guide indicates 97% (*n* = 7) RCY, which is achieved in the absence of added metal ions. For most values, error bars representing standard deviation are smaller than the symbols. For a full list of mean RCY (*n* = 3) and standard deviation values see Table SI-4.†

The results of competition experiments with THP and ^68^Ga^3+^ in the presence of a single spiked metal ion (at metal ion concentrations of 50 nM to 500 μM, THP concentrations of 5 μM, pH 7 in carbonate solution) are shown in Fig. 4 and summarised in Table SI-2.† In the absence of a metal ion spike, the RCY of [^68^Ga(THP)] was consistently ≥97%. The presence of Ni^2+^, Zn^2+^, Pb^2+^ and Cr^3+^ had no effect on ^68^Ga radiolabelling efficiency, even at metal ion concentrations 100-fold greater than THP concentration.

As expected, the presence of ^nat^Ga^3+^ reduced RCY of [^68^Ga(THP)] at ^nat^Ga^3+^ concentrations ≥5 μM (to 81 ± 0.78% at 5 μM, 9 ± 0.87% at 50 μM, and 0% at 500 μM), but no significant changes were observed at concentrations <5 μM. Similarly, at concentrations ≥5 μM, Fe^3+^, Al^3+^ and Ti^4+^ all reduced RCY of [^68^Ga(THP)]. At metal ion concentrations equimolar with THP, RCY of [^68^Ga(THP)] was 88 ± 3.81% for Fe^3+^ solutions, 93 ± 2.30% for Al^3+^ solutions and 87 ± 2.86% for Ti^4+^ solutions. At 50 μM metal ion concentrations, RCY of [^68^Ga(THP)] was 26 ± 1.25% for Fe^3+^ solutions, 79 ± 0.47% for Al^3+^ solutions and 62 ± 1.53% for Ti^4+^ solutions.

### Effect of ascorbate on radiolabelling THP in the presence of Fe^3+^

**Fig. 5:**
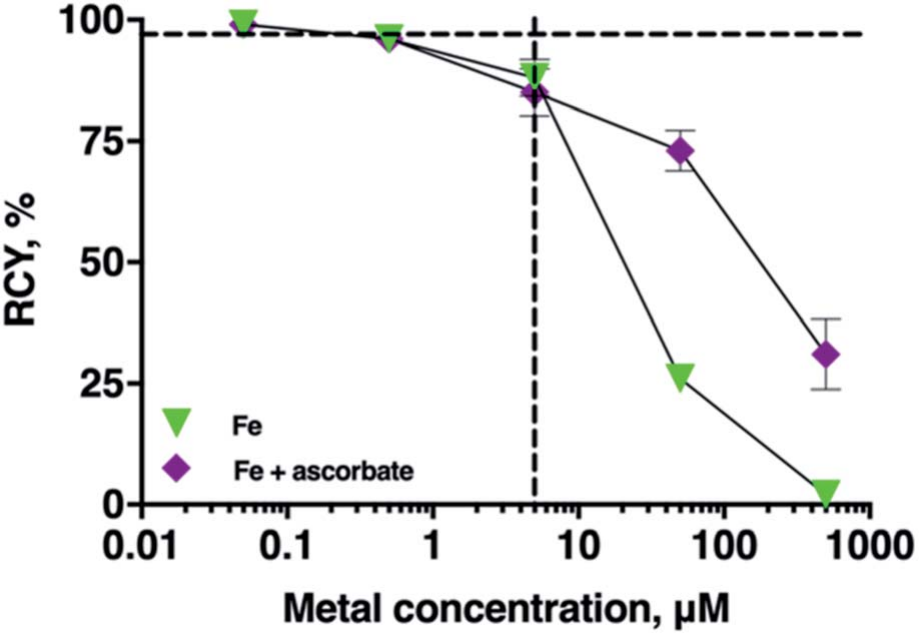
Mean RCYs of [^68^Ga(THP)] in the presence of either increasing concentrations of Fe^3+^ (green triangles) or ascorbate (166 mM) and increasing concentrations of Fe^3+^ (*n* = 5). The vertical dashed line indicates equimolar concentrations of chelator and metal ion (5 μM), and the horizontal dashed guide indicates 96 ± 0.4% (*n* = 6) RCY in the absence of spiked Fe^3+^. For most values, error bars representing standard deviation are smaller than the symbols.

In many radiopharmaceutical formulations, ascorbic acid or sodium ascorbate is included to minimise radiolysis of the solution. The presence of sodium ascorbate (166 mM, THP concentrations of 5 μM, and 5–7 MBq ^68^Ga, pH 7 in bicarbonate solution) in THP radiolabelling reactions did not reduce radiolabelling efficiency of THP with ^68^Ga (RCY 96 ± 0.4%, *n* = 6). Ascorbate can reduce Fe^3+^ to Fe^2+^. To assess the practical implications of this, the RCY of [^68^Ga(THP)] in reactions containing ^68^Ga^3+^, THP, a spike of Fe^3+^ and ascorbate were compared with the RCY of reactions containing ^68^Ga, THP and a spike of Fe^3+^ only (Fig. 5). In reactions containing Fe^3+^ at concentrations of 5 μM, addition of ascorbate did not affect RCY of [^68^Ga(THP)]. In reactions in which Fe^3+^ was present at concentrations of 50 or 500 μM, the presence of ascorbate resulted in increased RCY of [^68^Ga(THP)]: at [Fe] = 50 μM, RCY increased from 26 ± 1.25% to 73 ± 4.17%, and at [Fe] = 500 μM Fe, RCY increased from 2 ± 1.99% to 31 ± 7.29%.

### Radiolabelling THP with cation-exchange processed ^68^Ga

Numerous clinical ^68^Ga-radiolabelling protocols include eluate pre-processing steps in which ^68^Ga is isolated on a cation exchange cartridge, followed by a washing step and subsequent elution of ^68^Ga from the cartridge. These methods concentrate ^68^Ga and isolate ^68^Ga from (i) non-radioactive metal ion impurities (such as Fe, Ti and Zn) and (ii) ^68^Ge that is often present, albeit in very low amounts, in generator eluate containing high concentrations of HCl. There are three well-used methods that employ solutions of different composition to wash and to release ^68^Ga from the cartridge: the “NaCl method”, in which aqueous ^68^Ga solutions contain 5 M NaCl and 0.13 M HCl,^33,38^ the “ethanol method”, in which ^68^Ga solutions contain 90% ethanol and 0.9 M HCl,^9^ and the “acetone method”, in which ^68^Ga solutions contain 97.6% acetone and 0.05 M HCl.^10^

The reactivity and speciation of ^68^Ga in these mixtures with organic solvents could have effects on radiolabelling efficiency. To assess the tolerance of THP radiolabelling with pre-processed ^68^Ga in the presence of other components in solution such as NaCl, ethanol and acetone, we reacted THP with solutions of ^68^Ga processed according to these three methods. In these aqueous reaction solutions (final volume 300 μL) containing pre-processed ^68^Ga eluate (7–19 MBq, 50 μL, containing either ethanol, acetone or NaCl eluate solutions), the final THP concentration was 5 μM or 0.5 μM, and pH = 7.

We compared the RCY of these reactions with the RCY of reactions using unprocessed ^68^Ga (eluate straight from the generator). At 5 μM THP concentration, the RCY of [^68^Ga(THP)] was unaffected by using ^68^Ga eluent processed using either the NaCl method or the ethanol method, with RCY >96% (Table 1). At 0.5 μM THP concentration, the RCY was unaffected for reactions containing ^68^Ga eluent processed using the ethanol method, and slightly improved for reactions containing ^68^Ga eluent processed using the NaCl method (Table 1). In contrast, in reactions containing ^68^Ga eluent processed using the acetone method, the RCY of [^68^Ga(THP)] was significantly decreased at both THP concentrations of 5 μM and 0.5 μM.

**Table 1:** RCYs for the reaction of THP with unprocessed and pre-processed ^68^Ga^3+^ eluents. Radiolabelling experiments were undertaken in triplicate

	THP, μM	RCY ± SD (*n* = 3), %
Unprocessed ^68^Ga	5.0	96 ± 0.22
	0.5	59 ± 3.16
NaCl method	5.0	96 ± 0.58
	0.5	70 ± 0.30
Ethanol method	5.0	96 ± 0.35
	0.5	58 ± 3.33
Acetone method	5.0	92 ± 3.03
	0.5	31 ± 10.26

## Discussion

Previous studies^12,13,27–29^ have shown that THP and hydroxypyridinones have particularly high affinity for hard, oxiphilic cations such as Al^3+^, Fe^3+^ and Ga^3+^, with markedly lower affinity for divalent metal ions such as Zn^2+^ and Cu^2+^. Although THP has shown remarkably high affinity for Ga^3+^ (log *K*_1_ = 35.0, and pGa = 30.0 at pH 7.4) and quantitative RCYs when reacted with ^68^Ga^3+^ at chelator concentrations as low as 0.5 μM, trace metal impurities, particularly hard, oxiphilic metal ions, have a potential to compete with ^68^Ga^3+^ for complexation to THP.^12,13,19^ For example, for [Fe(THP)], log *K*_1_ = 34.2, and *p*_M_ = 29.1 at pH 7.4.^13^

Our competition experiments showed that the presence of Al^3+^, Fe^3+^, ^nat^Ga^3+^ and Ti^4+^ reduces RCY of [^68^Ga(THP)] at concentrations equimolar with THP and higher. Analytical ICP-MS studies indicate that all of these metals are present in generator eluate, and that the amounts can vary significantly with (i) the time between generator elutions and (ii) the age of the generator. These findings have implications for design of kits that contain THP chelator bioconjugates. At present, the GalliProst™ kit contains 40 μg of THP-PSMA, and when reconstituted by simple addition of unprocessed ^68^Ga generator eluate, THP-PSMA is at a concentration of 5 μM. Here, the range of concentrations of Ti (1.17–1.51 μM), Fe (0.07–0.09 μM), ^nat^Ga (0.04–0.21 μM) and Al (0.61–0.91 μM) observed in eluate are all <5 μM. Our experience to date^5,16^ shows that the presence of these metals does not affect ^68^Ga radiolabelling using GalliProst™ kits. However, if lower amounts/concentrations of THP bioconjugates were to be used, the combination of Al, Fe, Ga and Ti in (unprocessed) ^68^Ga eluate could decrease RCY of ^68^Ga radiolabelling in such kits.

^68^Ga radiolabelling of THP is less susceptible to the presence of some metal ions (Zn^2+^, Ni^2+^, Pb^2+^) compared to DOTA. Prior work has shown that the presence of a range of metal ions significantly decrease RCY of ^68^Ga-DOTA-based derivatives.^22,36,37^ For example, in ^68^Ga-radiolabelling reactions of DOTA-TATE, a 2 : 1 Pb^2+^ : DOTA-TATE molar ratio decreases RCY of ^68^Ga-DOTA-TATE to less than 80% (compared to near quantitative RCY in the absence of spiked metal ion). For Zn^2+^, a 6 : 1 ratio decreases RCY to below 80%. The presence of Zn^2+^ in ^68^Ga-radiolabelling reactions of NOTA also decreases RCY of [^68^Ga(NOTA)].^36^ Similar to the case of THP, RCYs of ^68^Ga-labelled DOTA and NOTA decrease in the presence of a molar excess of Fe^3+^ (relative to chelator). In contrast to THP, RCY of ^68^Ga-labelled DOTA and NOTA are less susceptible to the presence of Al^3+^.^36^

The multi-sample ICP-MS analysis reported here is (to date) the most comprehensive and robust quantification of metal ion impurities in the widely used E&Z ^68^Ga generator, although we note that eluates in this study were obtained from 12–18 months old E&Z ^68^Ga generators. Previous studies have quantified trace metal impurities in ^68^Ga generator eluate, including eluate from a Cyclotron Co. Ltd. generator (0.1 M HCl mobile phase, titanium dioxide solid phase, from Obninsk, Russia), a generator developed by ANSTO (Australia's Nuclear Science and Technology Organisation) and single eluate samples from E&Z ^68^Ga generators (0.1 M HCl mobile phase, titanium dioxide solid phase).^22,34,39^ The amounts of metal ion impurities in our multi-sample ICP-MS analyses are of a similar magnitude to previously reported data for eluate deriving from generators with a titanium dioxide solid phase.^14,22,34^ Our data on Al, Ti, Fe and Ga concentrations in eluates obtained six months apart from each other suggest that the concentration of these metal ions decreases over time/number of elutions.

We assessed whether the inclusion of other chemical components, such as ascorbate or organic solvents commonly used in ^68^Ga generator eluate pre-processing affect RCY of [^68^Ga(THP)]. Addition of ascorbate to ^68^Ga radiolabelling reactions did not result in decreased RCYs of [^68^Ga(THP)], and in this respect, it is a suitable radiolytic stabiliser for radiopharmaceuticals containing THP chelators. At Fe concentrations of 50 and 500 μM, the presence of ascorbate improved RCY of [^68^Ga(THP)], compared to reactions undertaken with a spike of Fe, but without ascorbate. We attribute this to Fe^3+^ reduction to Fe^2+^. Fe^2+^ has significantly lower affinity for hydroxypyridinones compared to Fe^3+^. Others' experimental data show that the rate of ascorbate reduction of Fe^3+^ to Fe^2+^ is pH dependent, and can decrease by an order of magnitude as the pH in aqueous media increases from 5 to 6.^40^ Over the timeframe of THP radiolabelling reactions (<5 min), and at Fe concentrations in generator eluate (<5 μM), ascorbate reduction of Fe^3+^ is unlikely to be of practical importance.

In some radiopharmacies, it has become standard practice to process ^68^Ga generator eluate, in order to separate ^68^Ga from ^68^Ge breakthrough. The presence of sodium chloride or ethanol in solutions of processed ^68^Ga eluate does not adversely affect RCY of [^68^Ga(THP)] compared to radiolabelling reactions in which unprocessed eluate is used, but the use of processed eluate containing acetone does result in a slight decrease in RCY and should be avoided in the context of THP-based radiopharmaceuticals.

### Concluding remarks

Metal impurities commonly present in ^68^Ga generator eluate can deleteriously affect chelator ^68^Ga radiolabelling of various bifunctional chelator-derived radiopharmaceuticals.^14,22,36,37^ We have provided experimental evidence that ^68^Ga radiolabelling of THP is less susceptible to the presence of some of these metal ions (Zn^2+^, Cr^3+^, Ni^2+^, Pb^2+^) compared to other clinically used chelators such as DOTA.

Other metal ions (Fe^3+^, Ti^4+^, Al^3+^, ^nat^Ga^3+^) can interfere with THP radiolabelling if present at sufficient concentration. However, the low concentrations at which these metals occur in generator eluate, combined with the amount of THP-based conjugate currently used in prefabricated kits, results in quantitative RCY of these ^68^Ga–THP conjugates. If lower amounts/concentrations of THP bioconjugates were used, the combination of Al^3+^, Fe^3+^, ^nat^Ga^3+^ and Ti^4+^ in unprocessed ^68^Ga eluate could decrease RCY of ^68^Ga radiolabelling in such kits, and kit design should take this into account.

## Conflicts of interest

There are no conflicts of interest to declare.

## Supplementary Material

RA-009-C9RA07723E-s001
