# Supplementary material for: The effects of trace metal impurities on Ga-68-radiolabelling with a tris(3-hydroxy-1,6-dimethylpyridin-4-one) (THP) chelator
Source: RSC Adv. 2019 Nov 14;9(64):37214–21. doi: 10.1039/c9ra07723e (PMC9075519; doi:10.1039/c9ra07723e)
Supplement: RA-009-C9RA07723E-s001 [file RA-009-C9RA07723E-s001.pdf]

Electronic Supplementary Material (ESI) for RSC Advances.
This journal is © The Royal Society of Chemistry 2019

The effects of trace metal impurities on Ga-68-radiolabelling with *tris*(3-hydroxy-1,6-dimethylpyridin-4-one) (THP) chelator

SUPPORTING INFORMATION

Ruslan Cusnir[1,2], Andrew Cakebread[3], Margaret S. Cooper[1], Jennifer D. Young[1], Philip J. Blower[1], Michelle T. Ma[1,*]

[1] School of Biomedical Engineering and Imaging Sciences, King's College London, St Thomas' Hospital, London SE1 7EH, United Kingdom

[2] Laboratory of Radiochemistry, Paul Scherrer Institute, 5232 Villigen-PSI, Switzerland

[3] Mass Spectrometry Facility, King's College London, Franklin Wilkins Building, 150 Stamford St, London SE1 9NH, United Kingdom

*michelle.ma@kcl.ac.uk

Table SI-1. Concentrations of selected trace metals in $^{68}Ga^{3+}$ eluate measured by ICP-MS. $^{68}Ga^{3+}$ was eluted from a $^{68}Ge/^{68}Ga$ E & Z generator with 0.1 M HCl from ABX GmbH with a pre-elution window of 2 h or 20-24 h.

Metal	0.1 M HCl blank		2 h pre-elution		1 day pre-elution	
	μM ± SD	n	μM ± SD	n	μM ± SD	n
Na	0.1787±0.2306	10	1.4076±1.1203	9	1.7901±0.6420	4
Al	0.2814±0.0538	10	0.6063±0.4674	9	0.9134±0.1602	4
Sc	0.0000±0.0001	10	0.0001±0.0000	9	0.0001±0.0000	4
Ti	0.0025±0.0006	10	1.1661±0.0579	9	1.1835±0.0239	4
V	0.0005±0.0005	10	0.0002±0.0007	9	0.0006±0.0012	4
Cr	0.0021±0.0012	10	0.0031±0.0012	9	0.0056±0.0028	4
Mn	0.0005±0.0001	10	0.0034±0.0037	9	0.0041±0.0007	4
Fe	0.0217±0.0135	10	0.0742±0.0471	9	0.0939±0.0195	4
Co	0.0003±0.0003	10	0.0005±0.0004	9	0.0008±0.0004	4
Ni	0.0035±0.0010	10	0.0056±0.0023	9	0.0054±0.0018	4
Cu	0.0021±0.0005	10	0.0029±0.0007	9	0.0034±0.0009	4
^{66}Zn*	0.0405±0.0450	10	0.0594±0.0370	9	0.0839±0.0195	4
^{68}Zn*	0.0241±0.0267	10	0.0405±0.0217	9	0.0853±0.0146	4
^{nat}Zn*	0.1453±0.1612	10	0.2128±0.1326	9	0.3008±0.0698	4
$^{decay68}Zn$*	-0.0031±0.0035	10	0.0006±0.0034	9	0.0289±0.0072	4
^{nat}Ga	0.0032±0.0015	10	0.0437±0.0200	9	0.2048±0.1199	4
^{72}Ge	0.0001±0.00004	10	0.0001±0.0001	9	0.0001±0.0001	4
Sn	0.0009±0.0006	10	0.0002±0.0002	9	0.0005±0.0007	4
Ba	0.0070±0.0024	10	0.0991±0.0457	9	0.4601±0.2758	4
Pb	0.0270±0.0292	10	0.1287±0.1623	9	0.4396±0.5605	4

*Concentrations of the individual isotopes, ^{66}Zn and ^{68}Zn, were determined by ICP-MS. ^{nat}Zn was calculated based on natural abundance of ^{66}Zn (27.9 %). ^{68}Zn arising from decay of ^{68}Ga ($^{decay68}Zn$) was calculated by subtracting naturally occurring ^{68}Zn (18.75 %) from ^{68}Zn determined by ICP-MS.

Table SI-2. Summary of metal concentrations (determined by ICP-MS) in ^{68}Ga eluates from an E&Z generator. P-values were calculated using multiple t-tests in Prism 7 software without correction for multiple comparisons.

Metal	Mean concentration in 0.1 M HCl "blank" samples (μM)	Mean concentration in samples collected with a 2 h pre-elution (μM)	Mean difference (μM)	p-value
Al	0.282±0.054	0.606±0.467	0.323	4.3×10^{-2}
Ti	0.00248±0.00062	1.167±0.0579	1.164	$<10^{-15}$
Fe	0.021±0.012	0.0744±0.0471	0.0534	3.05×10^{-3}
^{nat}Zn*	0.145±0.161	0.213±0.133	0.0676	0.336
$^{decay68}Zn$*	-0.0031±0.0035	0.000611±0.00345	0.00371	3.25×10^{-2}
^{nat}Ga	0.00317±0.00151	0.0437±0.02	0.0413	3.58×10^{-6}
Pb	0.0269±0.0292	0.129±0.162	0.103	6.67×10^{-2}

Metal	Mean concentration in 0.1 M HCl "blank" samples (μM)	Mean concentration in samples collected with a 1 d pre-elution (μM)	Mean difference (μM)	p-value
Al	0.282±0.054	0.915±0.16	0.633	6.88×10^{-8}
Ti	0.00248±0.00062	1.185±0.0239	1.183	$<10^{-15}$
Fe	0.021±0.012	0.0925±0.0195	0.0715	5.63×10^{-6}
^{nat}Zn*	0.145±0.161	0.3008±0.0698	0.156	9.28×10^{-2}
$^{decay68}Zn$*	-0.0031±0.0035	0.0289±0.0072	0.032	7.5×10^{-8}
^{nat}Ga	0.00317±0.00151	0.208±0.119	0.204	8.06×10^{-5}
Pb	0.0269±0.0292	0.438±0.56	0.411	3.04×10^{-2}

Metal	Mean concentration in samples collected with a 2 h pre-elution (μM)	Mean concentration in samples collected with a 1 d pre-elution (μM)	Mean difference (μM)	p-value
Al	0.606±	0.915±0.16	0.309	0.231
Ti	1.167±0.0579	1.185±0.0239	0.0183	0.566
Fe	0.0744±0.0471	0.0925±0.0195	0.0181	0.488
^{nat}Zn*	0.213±0.133	0.3008±0.0698	0.0879	0.244
$^{decay68}Zn$*	0.000611±0.00345	0.0289±0.0072	0.0283	8.34×10^{-7}
^{nat}Ga	0.0437±0.02	0.208±0.119	0.163	1.39×10^{-3}
Pb	0.129±0.162	0.438±0.56	0.308	0.145

*^{nat}Zn was calculated based on natural abundance of ^{66}Zn (27.9 %). ^{68}Zn arising from decay of ^{68}Ga ($^{decay68}Zn$) was calculated by subtracting naturally occurring ^{68}Zn (18.75 %) from ^{68}Zn determined by ICP-MS.

Table SI-3. Summary of metal concentrations (determined by ICP-MS) in ^{68}Ga eluates from a second E&Z generator, with samples obtained 6 months apart from each other. P-values were calculated using multiple t-tests in Prism 7 software without correction for multiple comparisons.

**Metal**	**12 months mean concentration (μM)**	**18 months mean concentration (μM)**	**Mean difference (μM)**	**p-value**
Al	0.445±0.129	0.306±0.045	0.139	6.11×10^{-2}
Ti	1.509±0.0911	0.919±0.0493	0.59	1.83×10^{-5}
Fe	0.0766±0.0195	0.0535±0.0201	0.023	0.165
^{nat}Ga	0.0639±0.0221	0.0145±0.0177	0.0494	1.27×10^{-2}
**Metals**	**0.1 M HCl blank 1 (12 months) (μM)**	**0.1 M HCl blank 2 (18 months) (μM)**	**Mean difference (μM)**	**p-value**
Al	0.410±0.0158	0.319±0.0192	0.0907	0.298
Ti	0.00235±0.000782	0.00301±0.00029	0.00065	0.176
Fe	0.0163±0.00191	0.0355±0.00645	0.0192	1.27×10^{-3}
^{nat}Ga	0.000999±0.00011	0.00458±0.000278	0.00358	4.32×10^{-7}
**Metal**	**0.1 M HCl blank 12 months**	**12 months mean concentration (μM)**	**Mean difference (μM)**	**p-value**
Al	0.410±0.0158	0.445±0.129	0.0348	0.769
Ti	0.00235±0.000782	1.509±0.0911	1.506	4×10^{-7}
Fe	0.0163±0.00191	0.0766±0.0195	-0.0603	1.42×10^{-3}
^{nat}Ga	0.000999±0.00011	0.0639±0.0221	-0.0628	2×10^{-3}
**Metal**	**0.1 M HCl blank 18 months**	**18 months mean concentration (μM)**	**Mean difference (μM)**	**p-value**
Al	0.319±0.0192	0.306±0.045	0.0136	0.593
Ti	0.00301±0.00029	0.919±0.0493	0.916	2.92×10^{-9}
Fe	0.0355±0.00645	0.0535±0.0201	0.0181	0.132
^{nat}Ga	0.00458±0.000278	0.0145±0.0177	0.0099	0.306

Table SI-4. Radiochemical yields (%, ± standard deviation) for the reaction of THP (5 μM) with $^{68}Ga^{3+}$ in the presence of progressively increasing concentrations of selected metal ions. Competition experiments were undertaken in quintuplicates (n=5 technical replicates) and reproduced three times (n=3 experimental replicates).

**Metal**	**Concentration, μM**	**RCY ± SD, %**
Al^{3+}	0*	97±0.80
	0.05	97±1.66
	0.5	95±0.37
	5	93±2.30
	50	79±0.47
	500	67±2.64
Ti^{4+}	0*	97±0.70
	0.05	97±0.46
	0.5	95±0.73
	5	87±2.86
	50	62±1.53
	500	6±1.22
Cr^{3+}	0*	98±0.06
	0.05	99±0.23
	0.5	96±0.09
	5	99±0.13
	50	97±0.05
	500	98±0.86
Fe^{3+}	0*	97±0.50
	0.05	99±0.37
	0.5	96±0.96
	5	88±3.81
	50	26±1.25
	500	2±1.99
Ni^{2+}	0*	97±0.11
	0.05	99±0.35
	0.5	95±1.28
	5	99±0.24
	50	96±0.54

	500	98±0.19
Zn^{2+}	0*	98±2.13
	0.05	99±0.63
	0.5	96±0.90
	5	99±0.24
	50	96±0.68
	500	99±0.31
$^{nat}Ga^{3+}$	0*	97±0.96
	0.05	98±2.21
	0.5	96±0.38
	5	81±0.78
	50	9±0.87
	500	0±0.25
Pb^{2+}	0*	96±1.37
	0.05	99±0.43
	0.5	96±2.08
	5	99±0.26
	50	96±0.71
	500	98±0.56

*This experiment measured radiochemical yield in the absence of a metal ion “spike”.
